# A case report: co-occurrence of probable Vogt-Koyanagi-Harada disease and diabetic retinopathy

**DOI:** 10.1186/s12886-024-03410-z

**Published:** 2024-04-02

**Authors:** Huan Li, Zhiyong Li, Ailing Mao, Ping Dong, Wei Wang

**Affiliations:** Hebei Provincial Key Laboratory of Ophthalmology, Hebei Provincial Clinical Research Center for Eye Diseases, Hebei Eye Hospital, No.399, Quanbei East Street, 54000 Xingtai, Hebei Province China

**Keywords:** Probable Vogt-Koyanagi-Harada disease, Diabetes retinopathy, Exudative retinal detachment, Choroidal detachment

## Abstract

**Background:**

Bilateral retinal detachment and choroidal detachment in a patient are rare occurrences. The presence of bilateral diabetic retinopathy (DR) in such a case is even rarer and complicates the condition.

**Case presentation:**

In this study, we document a case of unconventional VKH. Manifestations in this patient included intense peripheral retinal detachment and choroidal detachment, along with vitreous opacities akin to cotton wool spots, concurrent with DR. The diagnosis was considered as probable VKH with DR. Treatment according to VKH protocols, including high-dose corticosteroids, yielded positive results.

**Conclusions:**

VKH can co-occurrence with DR. VKH manifestations vary, and early, aggressive, and long-term treatment is essential. The complexity of treatment increases with concurrent DR, necessitating the use of immunosuppressants.

## Background

Bilateral retinal and choroidal detachments in a patient are uncommon. Conditions such as Vogt-Koyanagi-Harada disease (VKH) and sympathetic ophthalmia are common causes. The coexistence of bilateral diabetic retinopathy (DR) is exceedingly rare and adds complexity to the condition.

VKH typically presents with fever and headache in its early stages. Certain patients display symptoms of meningeal irritation including nausea, vomiting, and neck stiffness. These symptoms often escalate to loss of vision in both eyes, widespread chorioretinitis accompanied by serous detachment of the retina, followed by depigmentation of the choroid in advanced stages. During this phase, Fluorescence Fundus Angiography (FFA) reveals numerous initial hyperfluorescent spots, progressing to extensive ‘lake-like’ fluorescein pooling in later stages. Nonetheless, many patients display atypical clinical presentations in real-world scenarios.

## Case presentation

**Fig. 1:**
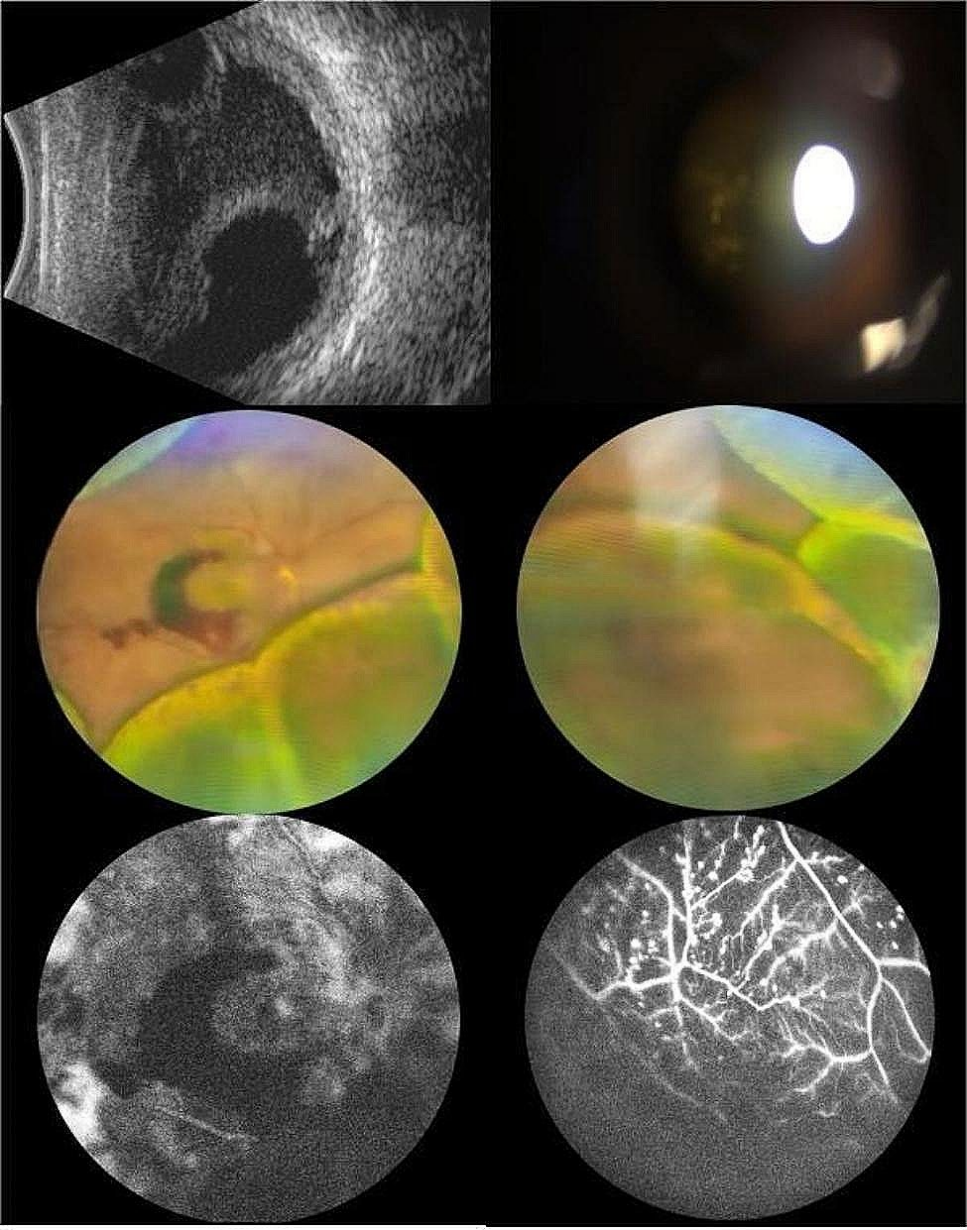
Eye condition of the patient on admission. **a**: B ultrasound: vitreous opacity, choroidal detachment and retinal detachment in both eyes. **b**: Glass white pompon-like cloudy. **c**: Fundus photography of the right eye. **d**: Fundus photography of the right eye. **e**: posterior pole FFA. **f**: peripheral imeter FFA

A 44-year-old Chinese man presented with a 45-day history of diplopia. Before the onset of his condition, he reported no cold, nausea, dizziness, tinnitus, vomiting, headache, hearing impairment, or hair graying. Local hospital assessments previously identified panuveitis, choroidal detachment, DR, and bilateral macular edema. Macular OCT indicated both eyes had macular neuroepithelium thickening and elevation, along with intercystic low reflex and localized detachment of the neuroepithelium. Despite initiating treatment with corticosteroid eye drops and posterior subtenon injections of corticosteroids, his condition deteriorated progressively. The patient reported no prior trauma or eye surgery. He had managed type 2 diabetes for 15 years and received bilateral total retinal photocoagulation for DR two years earlier. Physical exam revealed BCVA: manual in both eyes, with intraocular pressure at 17 mmHg and 18 mmHg. Exam findings were bilateral conjunctival congestion, atrial flash (+), localized posterior iris synechiae, pupillary margin neovascularization, clear lens, grade II vitreous opacity, and white pompon-like opacities. Right eye fundus examination showed a disc-surrounding neovascular membrane, 360° peripheral retinal and choroidal bulges, dispersed old photocoagulation marks, neovascularization, and superior and inferonasal retinal hemorrhages, extending to the posterior pole. In both eyes, examination revealed the presence of circumferential peripheral retinal and choroidal protrusions. These were accompanied by scattered, pre-existing photocoagulation marks. UBM detected a detachment of the ciliary body in each eye. Utilizing B-mode ultrasonography, vitreous opacity, and detachments in the choroid, and retina were noted for both eyes. The axial length of the right eye is 22.68 mm, and that of the left eye is 22.50 mm.Furthermore, OCT identified a significant bulge in the peripheral retina and a lack of clarity in the macular area. FFA revealed twisted, enlarged retinal veins, pronounced dotted fluorescence, and hemorrhagic fluorescence shading in the retina, with patchy nonperfusion areas (refer to Fig. 1). Orbital MRI and liver and kidney function tests were normal. Syphilis, HIV, and T-SPOT tests were negative. Intraocular fluid analysis ruled out microbial infection, with VEGF at 1614.3 pg/mL, BFGF at 478.3 pg/mL, IL-6 at 566 pg/mL, VCAM at 21845.2 pg/mL, and IL-8 at 135.7 pg/mL. HLA-DRB1 results indicated DRB1*04

**Fig. 2:**
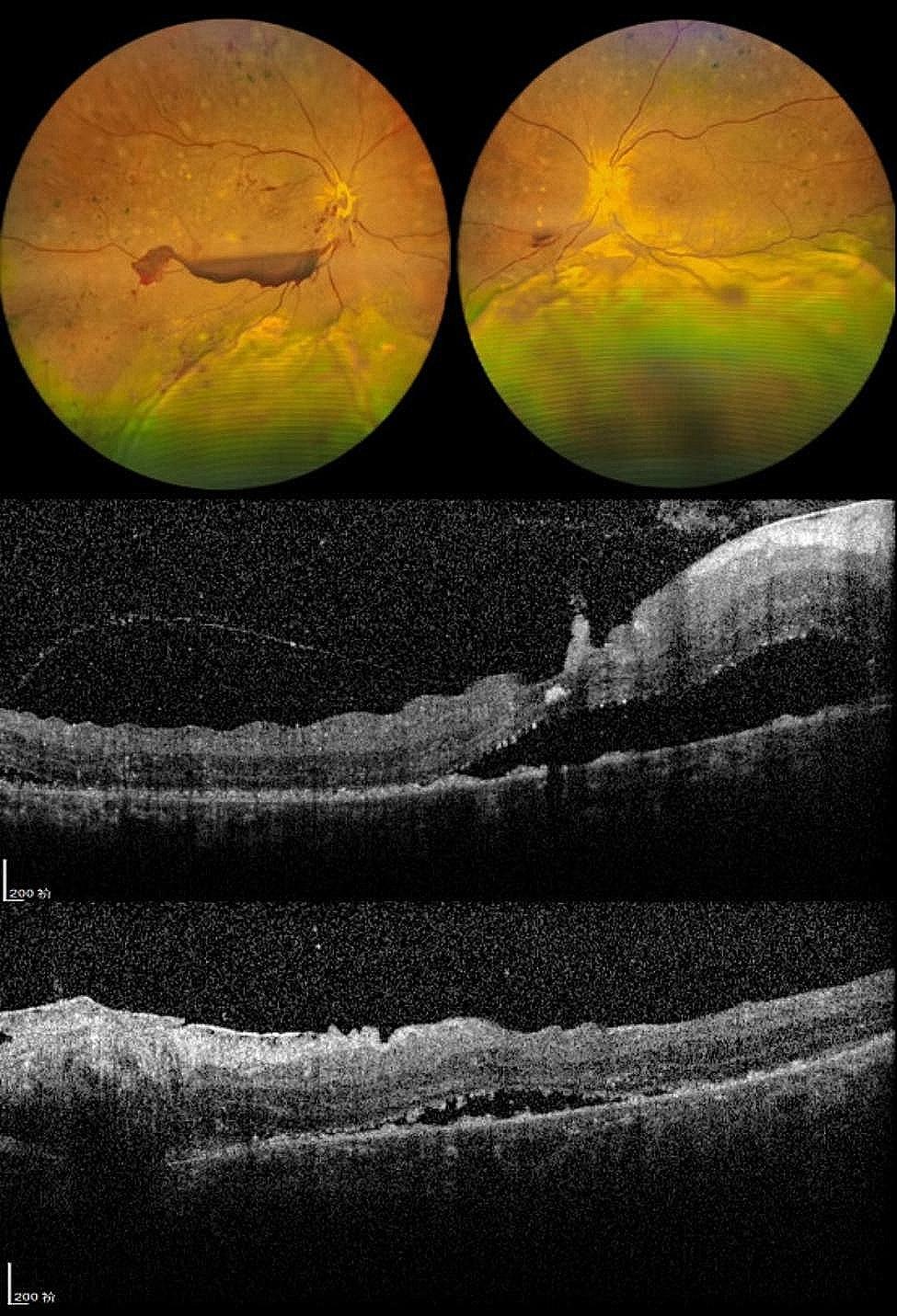
Eye condition of the patient at discharge. **a**: fundus photography of both eyes. **b**: macular OCT of the right eye. **c**: macular OCT of the left eye

**Fig. 3:**
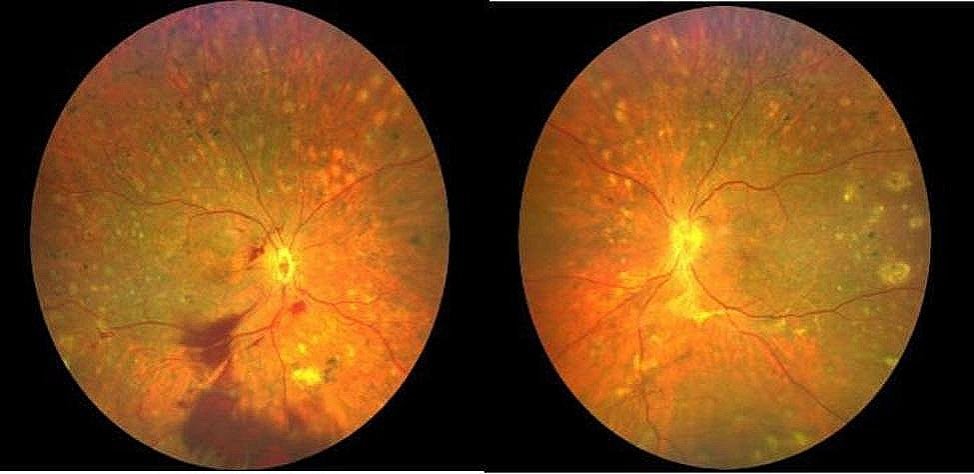
Fundus photography of both eyes at reexamination

He was therefore diagnosed with probable VKH. He was administered intravenous methylprednisolone at 0.5 g per day, gradually transitioning to oral corticosteroid therapy. Subsequently, the patient’s subretinal fluid resolved, and normal choroidal and retinal architecture was restored. Upon discharge, the BCVA was 20/250 in the right eye and 20/500 in the left eye (refer to Fig. 2). Post-discharge, despite several adjustments, the patient’s blood glucose levels remained poorly controlled. Consequently, oral cyclosporine at 100 mg per day was added to his regimen. However, the patient experienced general weakness and dizziness, leading to the discontinuation of cyclosporine after 12 days. At the follow-up examination, there was an improvement in retinal and choroidal detachments, with a BCVA of 20/200 in both eyes. Nonetheless, fundus hemorrhage in the right eye had worsened (refer to Fig. 3). The patient was then switched to cyclosporine from different manufacturers.

## Discussion and conclusion

VKH frequently presents with exudative retinal detachment. Other etiologies encompass sympathetic ophthalmia, serpiginous choroiditis, uveitis due to syphilis, sarcoidosis, posterior scleritis, tuberculosis, uveal effusion syndrome, and intraocular lymphoma. Nearly half (47.4%) of VKH cases develop bilateral exudative retinal detachment. Conversely, diagnosed non-VKH patients tend to have a greater incidence of unilateral exudative retinal detachment compared to those with VKH [1–2].

VKH, an autoimmune condition, is marked by bilateral granulomatous uveitis, frequently accompanied by meningeal symptoms, auditory issues, and abnormalities in skin and hair. Most commonly, it affects individuals aged 20 to 50 without major gender differences [3]. Moorthy’s 1995 classification of VKH outlined four stages: prodromal, acute uveitis, chronic/recovery, and chronic relapse. The disease presents variedly, with patients not necessarily undergoing all four stages. Early, precise diagnosis and treatment are crucial to avert progression to anterior uveal involvement and repeated attacks of granulomatous anterior uveitis. Internationally recognized, the revised VKH diagnostic criteria from the 2001 American Journal of Ophthalmology [4] divide the disease into three types: complete, incomplete, and probable.

Early VKH diagnosis is contingent on specific criteria: (1) No prior history of penetrating eye trauma or surgery before uveitis development. (2) Absence of signs or laboratory findings pointing to alternative eye disorders. (3) Involvement of both eyes. (4) Either observed diffuse choroiditis and exudative retinal detachment or, lacking overt symptoms, diagnostic imaging such as OCT or ultrasound indicating choroidal thickening, retinal exudative detachment, early hyperfluorescent leakages on FFA, and delayed subretinal fluorescein pooling [5].

The patient had a bilateral presentation of the condition, with no prior ocular trauma or surgery, and lacked signs of infectious uveitis, systemic rheumatic illness, or any other eye diseases. This case was atypical for VKH due to missing prodromal signs and the unusual occurrence of vitreous opacities similar to cotton wool spots, yet severe exudative retinal detachment was evident initially. The neuroepithelial detachment in the macula observed early on did not suggest macular edema. Peripheral choroidal detachment is a common early finding in VKH [6]. The widespread periphery of the exudative retinal detachment likely stemmed from prior bilateral retinal photocoagulation, which hindered subretinal fluid build-up in the posterior segment. The extensive detachment limited the efficacy of OCT and FFA diagnostic procedures. Inadequate corticosteroid administration at the initial treatment stage in the local hospital led to uncontrolled inflammation. The patient’s HLA-DRB1 test identified the DRB1*04 allele, a potential genetic indicator for VKH, particularly in the Han Chinese demographic [7–8].

Consequently, we diagnosed the patient with probable VKH disease. Hormonal shock therapy yielded positive results, stabilizing the retinal and choroidal detachments and improving visual acuity. However, the patient’s diabetic retinopathy continued to progress, with increased vitreous blood in the right eye. Some researchers advocate combining immunosuppressants and antimetabolites, using cyclosporine and biologics as first-line treatments for VKH to minimize complications and recurrences.

The concurrent occurrence of VKH and diabetic retinopathy, while uncommon, is a possibility [9]. Atypical VKH may present with vitreous opacities that resemble cotton wool spots, along with significant retinal and choroidal protrusion and peripheral retinal detachment. In instances of concurrent diabetic retinopathy, it is crucial to rigorously monitor blood glucose levels, considering the potential aggravation of the condition due to inflammation [10]. Consideration should be given to additional retinal photocoagulation, along with immunosuppressive therapy and, where needed, intravitreal injections of anti-VEGF agents or corticosteroids.

## Data Availability

All the data were included in the manuscript.
